# Supplementary material for: The Discrete and Continuous Retardation and Relaxation Spectrum Method for Viscoelastic Characterization of Warm Mix Crumb Rubber-Modified Asphalt Mixtures
Source: Materials (Basel). 2020 Aug 23;13(17):3723. doi: 10.3390/ma13173723 (PMC7503532; doi:10.3390/ma13173723)
Supplement: Supplementary file 1 [file materials-13-03723-s001.pdf]

Table S1 Raw data on dynamic modulus and phase angle for types of mixture

temperature/℃	Data<br>raw data of complex test experimental		
	**frequency/Hz**	**dynamic modulus/Mpa**	**phase angle/°**
5	25	16689	14.41
	20	16242	14.81
	10	14772	16.26
	5	13236	17.89
	1	9773	22.12
	0.5	8258	23.99
	0.1	4955	28.52
20	25	7557	26.21
	20	7060	26.30
	10	5778	27.91
	5	4642	29.33
	1	2604	32.33
	0.5	1996	32.35
	0.1	1062	31.43
35	25	3090	29.46
	20	2838	29.33
	10	2137	30.03
	5	1629	30.49
	1	844.3	30.07
	0.5	682.5	28.89
	0.1	436.8	26.43
50	25	969.7	32.80
	20	870.8	31.08
	10	641.9	30.02
	5	457.7	29.87
	1	244.9	27.09
	0.5	220.3	23.55
	0.1	177.1	21.50
